# Cross‐national patterns of behavioral and psychological symptoms of dementia in long‐term care facility residents: Integrating data from Japan and United States

**DOI:** 10.1002/alz70858_102666

**Published:** 2025-12-26

**Authors:** Asuna Arai, Yuriko Katsumata

**Affiliations:** ^1^ Faculty of Medicine and Graduate School of Medicine, Hokkaido University, Sapporo, Hokkaido, Japan; ^2^ Department of Biostatistics, College of Public Health, University of Kentucky, Lexington, KY, USA; ^3^ Sanders‐Brown Center on Aging, University of Kentucky, Lexington, KY, USA

## Abstract

**Background:**

Preliminary analyses with data from Japan and the United States separately suggest that the longitudinal patterns of behavioral and psychological symptoms of dementia (BPSD) are similar across both countries. This study aims to confirm these trajectory patterns and identify the associated factors using integrated data from Japan and the United States.

**Method:**

Data on individuals residing in long‐term care facilities were collected and integrated over four waves since 2015 from cohort studies conducted annually in Japan and the United States. In the United States, data were provided by the National Alzheimer's Disease Coordinating Center (NACC) and included two categories of long‐term care facilities: the assisted living, adult family homes, or boarding homes, and skilled nursing facilities, nursing homes or hospitals. In Japan, long‐term care facilities were classified as adult group homes for people with dementia, long‐term care health facilities, and intensive care homes for the elderly. BPSD were assessed using Neuropsychiatric Inventory in both countries. Group‐based multi‐trajectory modeling was employed to identify longitudinal patterns across 12 BPSD symptoms. Multinomial logistic regression analysis was used to examine factors associated with membership in each trajectory group.

**Result:**

A total of 644 residents from long‐term care facilities were included in the analysis. Four distinct BPSD trajectory groups were identified: Low, Agitation/Irritability Predominant, Depression/Anxiety Predominant, and High. Factors such as country, age, gender, marital status, cause of dementia, and the interaction between activities of daily living and cognitive function were found to be associated with the probability of group membership, with variations depending on the specific trajectory group.

**Conclusion:**

By integrating data from long‐term care facility residents in Japan and the United States, we identified four BPSD trajectory groups. Despite the limited overlap in variables between the two countries, we determined the associated factors for each trajectory group. These findings provide a foundation for Japan and the United States to collaboratively develop and implement BPSD prevention measures tailored to their long‐term care facility residents within a shared framework.

